# Auditable Clean-in-Place Decision Support from Routine SWRO SCADA: Selecting Differential-Pressure Recovery and Falsifying a Per-CIP-Reset Trigger

**DOI:** 10.3390/membranes16080257

**Published:** 2026-07-28

**Authors:** Yi Hsiang Su, Fan Cheng Meng, Pieh Yu Chang

**Affiliations:** Nedtex Technology Company, 5F, No. 112, Section 2, An Ho Road, Da’an District, Taipei 106, Taiwan

**Keywords:** seawater reverse osmosis, membrane cleaning, clean-in-place, estimand, decision support, identifiability, single-plant SCADA, techno-economics

## Abstract

Reverse osmosis (RO) desalination operators time membrane clean-in-place (CIP) by non-site-calibrated vendor thresholds. We present an auditable workflow that converts these thresholds into plant-calibrated decision support. It pre-specifies the recovery target and analysis unit, compares candidate signals with a five-test label-free battery, and reports composite weights only when identifiable. Using ≈two years of routine 10 min SCADA from one three-train island seawater RO plant (≈600 m^3^ d^−1^), the battery selects normalised feed channel differential pressure (DP_norm) for site-specific cleaning-recovery review. On the false discovery rate (FDR)-effective unit (*n* = 14 campaigns), recovery is marginal and not FDR-significant (mid-*p* Benjamini–Hochberg q ≈ 0.141); DP_norm is therefore an operator-review signal, not an autonomous or FDR-confirmed trigger. The same battery invalidates a per-CIP-reset net driving pressure trigger as a clip-floor regression-to-the-mean artefact; it collapses under the pre-specified matched placebo and is reproduced by a fake-date null, a finding that is bounded to this plant and estimand, and not causal proof of a unique mechanism. The DP_norm of ≥1.20 review point is an exploratory, in-sample heuristic selected partly against the endogenous operator log, and its indexed economic comparison inherits that limitation. An identifiability-gated Bayesian power prior leaves the four-weight composite non-identifiable on this single-regime plant, pinning only the near-zero salt passage weight. The contribution is a bounded, estimand-based workflow that supports audit without replacing operators or the safety envelope.

## 1. Introduction

Operators of seawater reverse osmosis (SWRO) plants arrest membrane fouling and scaling by clean-in-place (CIP) chemical cleaning, but the decision of when to clean often follows generic manufacturer criteria rather than a site-calibrated quantitative criterion. At this site, the bulletin specifies cleaning when any of these three normalised indicators crosses a threshold relative to start-up or the previous cleaning: permeate flow is down by 10%, salt passage is up by 10%, or feed-to-concentrate differential pressure is up by 15% [1]. Operators also judge whether a cleaning has helped. The indicators need not move together: differential pressure can respond before salt passage [2]. The method developed here compares candidate recovery signals from routine single-plant SCADA and reports what the record does and does not identify.

Two gaps follow. First, vendors supply single-indicator thresholds [1,3,4] but there are no objective composite fusing the hydraulic, productivity, and rejection channels into one defensible health index, and no transparent procedure for calibrating one to a plant’s own operating record. Second, although data-driven CIP studies show that learning cleaning policy from operating data can cut chemical use and extend run time [5,6], machine-learning fouling prediction [7], digital-twin restoration planning [8], and full-scale supervised fouling prediction at a seawater RO plant [9] are advancing rapidly; such demonstrations are typically single-facility, and single-plant studies are often viewed as limited in generalisability. The premise beneath that view (that the plant is the unit of analysis, so one plant is one sample) is the one we re-examine.

**Figure 1:**
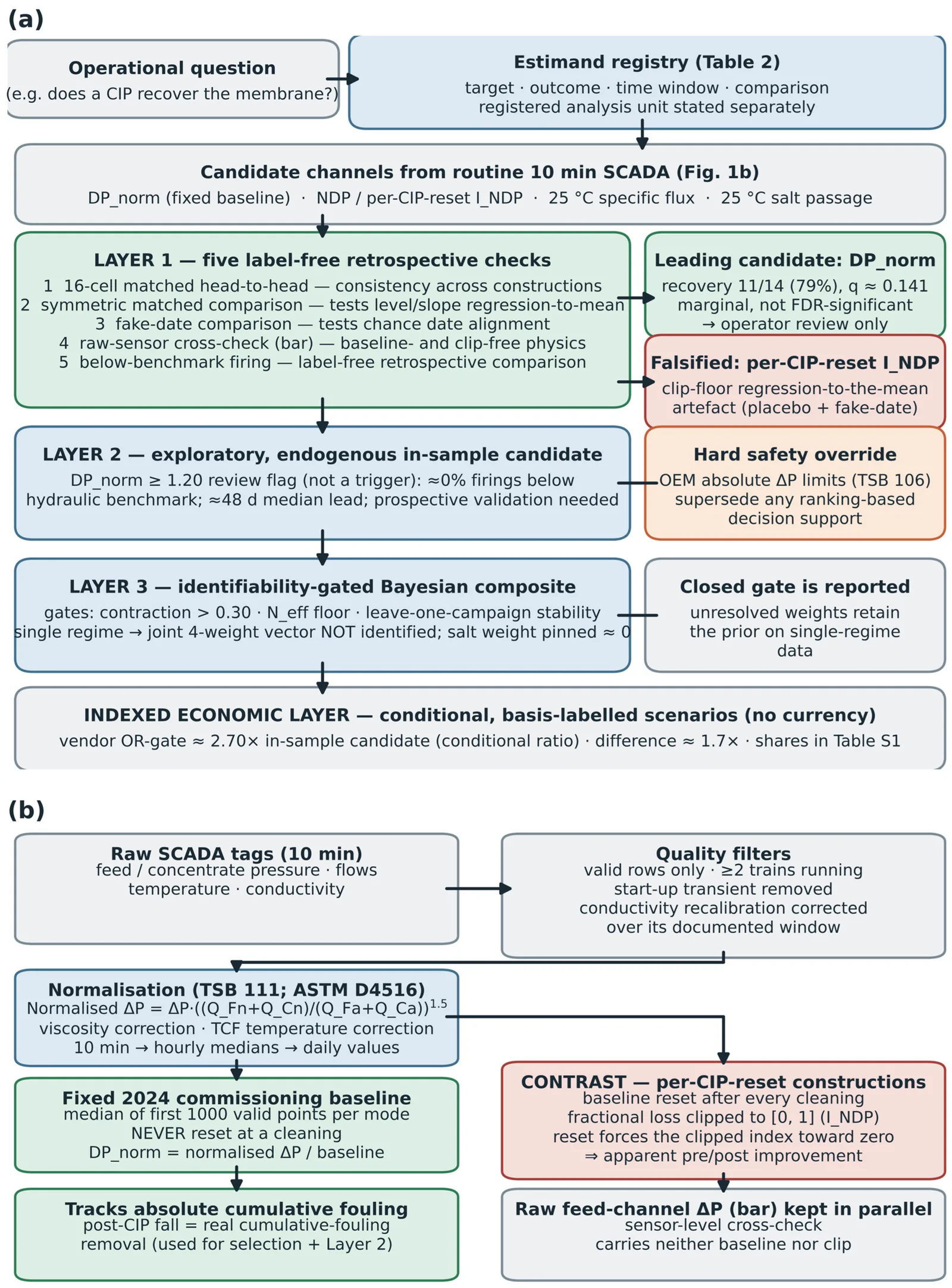
Estimand-registered, three-layer decision-support workflow. (**a**) The estimand defines the target; the registered campaign, event, day or train remains the analysis unit. Layer 1 compares recovery channels with five label-free retrospective checks. DP_norm is the leading operator-review candidate (11/14; mid-*p* BH q ≈ 0.141), whereas per-CIP-reset I_NDP is falsified on this record as a clip-floor artefact; it fails the matched placebo and fake-date checks, a pattern predicted by reset/clip effects. Layer 2 reports DP_norm ≥ 1.20 as an exploratory, endogenous, in-sample candidate. Layer 3 reports unresolved composite gates, and the economic layer gives conditional indexed scenarios. OEM absolute differential pressure limits remain a hard override [1]. (**b**) DP_norm is constructed from quality-filtered SCADA using TSB 111 flow normalisation, viscosity and temperature correction, daily aggregation and a fixed commissioning baseline [1,11]. Resetting a clipped per-CIP loss index toward zero can create apparent pre/post improvement. Raw feed channel ΔP is retained as baseline- and clip-free, but sensor-dependent and cross-check.

Our reframing is that the estimand affects which indicator matters: the target defined by a precise operational question [10]. In plain terms, an estimand fixes what is to be estimated—outcome, time window and comparison—before any indicator is examined; the registry also declares the effective independent campaign or event that carries the analysis. This vocabulary helps prevent post hoc selection of whichever indicator, window and construction looks best. On the same plant and SCADA archive, the cleaning-recovery estimand selects normalised feed channel differential pressure (DP_norm), whereas the composite weight estimand leaves the joint four-weight vector non-identifiable and pins only the near-zero salt passage marginal (Figure 1). The estimand defines the target; it is not itself the unit of analysis. Repeated events at one well-instrumented facility support site-specific estimation, but they are neither independent plants nor evidence of cross-site generalisability.

The resulting contribution is a three-layer methodology for site-specific CIP decision support (Figure 1); the empirical DP_norm finding illustrates it. Layer 1 tests candidate recovery signals without operator effectiveness labels, which would otherwise feed the operators’ pressure-based decisions back into the comparison. DP_norm is directionally consistent, but recovery on the FDR-effective unit is marginal and not FDR-significant (11/14 campaigns; exact two-sided sign test *p* ≈ 0.057; mid-*p* Benjamini–Hochberg *q* ≈ 0.141), so it remains an operator-review signal. The per-CIP-reset NDP loss index (I_NDP) fails the matched placebo and fake-date checks. That pattern is consistent with a reset/clip-floor mechanism, in which an index that resets after cleaning and is floored at zero can appear to rebound, but the checks do not by themselves prove the mechanism causally.

Layer 2 reports DP_norm of ≥1.20 as an exploratory, in-sample review heuristic with a retrospective below-benchmark firing descriptor. Because the candidate was selected partly using concordance with the endogenous operator log, neither the threshold nor the economic layer that uses it is independently validated. Layer 3 gates a four-weight composite on identifiability; on this near-single-regime plant (cross-train cosine 0.937–0.999, excitation rank ≈ 1), only the near-zero salt passage marginal contracts. No membrane life-extension benefit or validated currency saving is claimed. OEM differential pressure safety limits remain a hard override [1], and “label-free” means that no operator effectiveness or manual fouling-state label enters Layer 1.

## 2. Materials and Methods

### 2.1. Study Site, Dataset and Anonymisation

The study corpus is the routine operating record of one small seawater RO (SWRO) facility on an offshore island. Production is split across three independent trains, labelled A, B and C, whose combined nameplate output is ≈600 m^3^ d^−1^ (≈25 m^3^ h^−1^ when all three run). Each train carries three pressure vessels loaded with eight elements apiece, so the installed fleet totals 72 elements drawn from a single commercial 8-inch seawater membrane class; under heavy fouling the lead element of this class has previously shown feed-side pressure drop rising by as much as +218% per year [12], the observation that motivates the hydraulic emphasis of the present analysis. Because routine-delivered demand sits below the full three-train nameplate, any two trains suffice to meet it; taking one train offline for a CIP is therefore covered by the other two together with treated-water storage, leaving net production essentially unaffected—a structural property of the plant on which the techno-economic model relies directly (Section 2.9). The record itself is a 10 min resolution SCADA archive of roughly 9.7 × 10^4^ ten-minute observations, running from 2024 to 2026 (about two years).

For reasons of operational confidentiality and ongoing legal proceedings, the plant owner—a public water utility—is not identified at any point in this work, and the site is described only to the minimum engineering resolution that reproducibility demands. Withheld alongside the owner’s identity are the procurement figures, the membrane element model and area, the exact calendar dates, and the raw SCADA stream. These steps lower the risk of reverse identification without rendering the site truly anonymous, because the authors cite their own related work on the same plant (Section 4.6); what they protect is therefore the owner’s identity together with every specific commercial and raw data. Three parties may still be named where a particular fact requires it: the membrane vendor (LG Chem, referenced solely through its public Technical Service Bulletins [1]), the Water Resources Agency, and Taipower. The operating party—Nedtex Technology, the company with which the authors are affiliated—appears only as the operations-and-maintenance (O&M) contractor, which constitutes the principal conflict of interest and is declared as such.

Here “label-free” has a narrow operational meaning: no operator verdict on cleaning effectiveness and no hand-entered fouling-state annotation enters Layer 1. The CIP ledger supplies only date, train and acid/alkaline chemistry; effects are recomputed from SCADA. Excluding effectiveness labels avoids introducing a feed pressure-dependent label, because operators already monitor feed channel pressure.

### 2.2. Byte-Exact Processing Pipeline and Normalised Membrane Indicators

All derived quantities are produced by one version-fixed processing pipeline (internally “009”), so every reported value traces to a re-runnable computation on the archived dataset. The fixed preprocessing sequence is: (i) feed conductivity correction (raw conductivity divided by 1.2116 over the documented calibration window); (ii) retention of intervals with at least two concurrently running trains; (iii) removal of the 30 min start-up transient; and (iv) per-train feature computation. Temperature correction uses the vendor coefficients (TCF_A = 3070 K, TCF_B = 6200 K). Missing or invalid constituent tags are excluded rather than imputed; hourly medians before daily aggregation reduce short spikes; and the documented conductivity recalibration is corrected over its recorded window. No independent pressure-transmitter zero/span history, redundant sensor check, or full pressure-calibration audit was available. The raw-sensor check removes baseline construction but is not drift-free, and the fake-date analysis does not identify gradual transmitter drift. Residual pressure-sensor drift therefore remains a limitation.

Membrane state is summarised by four normalised channels: net driving pressure (NDP), the viscosity-corrected normalised feed-to-concentrate differential pressure (DP_norm), 25 °C specific flux, and 25 °C normalised salt passage; normalisation follows the vendor’s data-normalisation equations (LG Chem TSB 111, pp. 28–32; normalised ΔP = ΔP·((Q_Fn + Q_Cn)/(Q_Fa + Q_Ca))1.5) [1], consistent with general RO performance data standardisation practice [11]; Figure 1b illustrates the full construction from raw tags to DP_norm. DP_norm is computed per operating mode (defined by the number of concurrent trains) against a fixed 2024 commissioning baseline (the median of the first 1000 valid points in each mode, with the three-train baseline excluding the commissioning period), and is never reset at a cleaning. Anchoring to a fixed baseline rather than a per-cleaning baseline is the primary ageing/drift control of this study: it makes DP_norm track absolute cumulative fouling, so that a post-CIP fall reads as cumulative fouling removal and not as a per-cleaning baseline reset. The un-normalised feed channel differential pressure (in bar) is retained in parallel as a sensor-level cross-check that carries neither baseline nor clip. The deposited derived daily table, the label-free ledger, the window definitions with fixed seeds, and the regeneration scripts (Data Availability Statement) reproduce every reported table and figure value, and a SHA-256 manifest pins the withheld raw series so that the upstream derivation can be audited under the confidential-access route. Separately, and only as inputs to the composite weighting (Section 2.8), each channel’s fractional loss relative to a per-CIP baseline (clipped to [0, 1]) is computed; these per-CIP-reset constructions (‘dp_pct’, ‘ndp_pct’; per-CIP-reset NDP loss is denoted I_NDP) are kept strictly distinct from the fixed-baseline DP_norm and are used only as like-for-like recovery diagnostics, never as the primary fouling state. All decision support is, finally, bounded below the OEM absolute differential pressure safety limits (TSB 106, p. 13: 1 bar per element and 3.8 bar per housing), which act as a hard safety override that supersedes any ranking-based decision support [1].

### 2.3. Label-Free CIP Ledger and Analysis-Unit Crosswalk

The objective CIP ledger yields 23 train-legs. Because most adjacent legs separated by 0–2 d are the acid and alkaline stages of a single cleaning, legs of the same train within ≤7 d are collapsed into one campaign (18 campaigns; A: 3, B: 8, C: 7), of which 14 carry complete pre- and post-cleaning windows. Campaigns on different trains within ≤3 d are then treated as one plant-wide event, leaving approximately 11 effective independent events. A separate label-free event-pair ledger of *n* = 17 underlies the head-to-head construction grid (Section 2.5).

Because these counts index different units of analysis, conflating them would misstate the evidence base. We therefore report them as a branched crosswalk (Table 1) in which each analysis is paired with its own unit definition and count: train-legs (23), within-train campaigns (18), complete-window campaigns (14), effective independent events (≈11), the event-pair ledger (*n* = 17), the 16-cell estimand grid, and the FDR-effective *n* ≈ 11–14. The crosswalk is explicitly not a monotone funnel: the *n* = 17 event-pair ledger, for example, is a distinct unit-specific view and not a subset step within the ≈11 effective-independent collapse. All multiplicity control and statistical-power statements are tied to the FDR-effective *n* ≈ 11–14, never to the 23 legs, whose pseudo-replication (overlapping windows, acid/alkaline pairing) would inflate significance and is reported as descriptive only.

### 2.4. Estimand Registry

Each operational question is registered (Table 2) by its outcome, time window, comparison, and unit of analysis, together with the channel that question selects or identifies on this plant and the evidential status of that finding in the present work. The registry holds two estimands, both native to this manuscript: the cleaning-recovery/operator decision-support estimand (E1 → DP_norm) and the Bayesian-identifiability estimand (E2 → salt passage weight pinned near zero). Other operational questions on the same record (for example short-horizon predictability for real-time monitoring) define further estimands that would in general still select other channels and are not analysed here. A semantic guard is stated once and held throughout: in E2, “the salt weight is identified” means its weight is pinned by the data to a near-zero value (small contribution), not that salt passage drives cleaning; the two estimand answers are two answers to two different questions, not co-equal drivers.

### 2.5. Layer 1: Label-Free Validation Battery

Layer 1 compares measured channels before a threshold or weight is tuned. It excludes operator effectiveness labels and per-CIP clip-based “benefit curves”. Such a curve self-correlates for both candidates: a floor-clip null with zero real recovery has mean Spearman ρ ≈ +0.66 (90% interval [0.43, 0.85]), so the observed per-CIP-reset I_NDP (+0.951) and fixed-baseline ‘dp_pct’ (+0.556) are not by themselves recovery evidence. Selection therefore rests on five checks whose results are reported separately.

For each candidate channel we compute, around each independent campaign, medians over [d_0_ − 14, d_0_ − 1] and [d_0_ + 1, d_0_ + 14] (≥3 valid points each). Cleanable Δ = (post − pre) − placebo, where the placebo is the median change in ≥300–600 random same-train windows centred ≥28 d from any CIP; this reduces sensitivity to slow baseline change and mean reversion but does not remove sensor drift. The five checks are: (i) recovery direction across a 16-cell construction grid; (ii) a symmetric matched placebo balancing pre-event level and slope; (iii) a fake-date comparison; (iv) un-normalised, un-clipped feed channel dP; and (v) below-benchmark firing, defined without operator dates.

### 2.6. Layer 2: Exploratory Operator-Review Candidate and Retrospective Below-Benchmark Firing

Layer 2 examines plant-specific review thresholds after channel selection. Daily DP_norm is smoothed with a causal trailing 7-day median; for τ ∈ {1.10, 1.15, 1.20, 1.25, 1.30}, contiguous days at or above τ (gaps ≤ 14 d merged) define a firing episode. Below-benchmark firing is reported as a targeting descriptor, not proof that a cleaning was unnecessary. The 1.20 candidate was chosen in-sample from the grid using a trade-off among operator-log precision, sensitivity, missed cleanings and duty cycle. Because operators already watch feed channel pressure, that selection is endogenous; its apparent performance may be inflated and requires prospective validation. The label-free below-benchmark descriptor is reported separately and does not make the selected threshold independent of the log.

### 2.7. Statistical Analysis

We use the exact binomial sign test, the Wilcoxon signed-rank test, and a sign-permutation test; unless stated, *p*-values are two-sided. Test selection is dictated by the small effective sample: exact nonparametric tests require no distributional assumption at *n* ≈ 11–14, the permutation and placebo procedures build their nulls directly from the record, and parametric alternatives were avoided because their assumptions are unverifiable at this n. For the primary complete-campaign inference, the sign-test *p*-value and BH *q*-value are reported with the recovery proportion and Wilson 95% CI; matched correlations, raw-sensor changes and firing fractions are descriptive magnitudes. Multiplicity is controlled across the four channels by the Benjamini–Hochberg false discovery rate (FDR) procedure [13] applied to the mid-*p* sign-test values; for DP_norm the exact two-sided sign test gives *p* ≈ 0.057 (mid-*p* ≈ 0.035), so the BH-adjusted value is *q* ≈ 0.141 (mid-*p* × 4 channels), recomputable from the deposited derived daily table and label-free CIP ledger using the pre/post sign-test procedure documented there. All power and FDR statements are tied to the FDR-effective unit (*n* ≈ 11–14), never to the 23 legs. Proportion confidence intervals use the Wilson method [14]. Random procedures use fixed seeds for reproducibility: placebo windows draw 300–600 random segments; complementarity nulls draw 300–500 simulations per seed and are reported across multiple seeds; weight stability uses 2000 bootstrap resamples. We flag explicitly that a secondary “coverage” diagnostic proved seed-sensitive on re-verification (an earlier single-seed value of *p* < 0.004 for DP-owned coverage was unstable and is *p* ≈ 0.05–0.14 across seeds); all conclusions rest on the stable primary results, not on that diagnostic.

### 2.8. Layer 3: Identifiability-Gated Bayesian Composite Weighting

The four-weight composite combines normalised NDP, DP_norm, flux and salt passage. Free regression on this single-regime record would produce unstable but precise-looking coefficients, so a Dirichlet-type, DP-dominant physics prior is updated through a power prior [15]. A coordinate moves from the prior only if posterior spread contracts by >0.30, its effective-independent campaign count clears the N_eff floor, and leave-one-campaign deletion is stable. Cross-train homogeneity is measured by leading-direction cosine similarity and weight stability by bootstrap argmax share. At N_eff < 8, no coordinate is released from the prior.

### 2.9. Economic Model

The per-CIP cost at this plant is dominated by chemicals (EDTA-dominant) and attended labour, with net production loss ≈ 0 because two of the three trains meet routine demand (Section 2.1). To avoid disclosing site-identifying procurement figures, all economic quantities are reported only as dimensionless ratios and multiples, never as absolute currency. Because the labour and logistics component can be scoped in more than one defensible way, every absolute-magnitude quantity (the per-CIP cash and its shares of annualised membrane replacement) is reported under two labelled costing bases—an accounting basis (chemicals plus single-operator attended labour) and a fuller full-operation basis (chemicals plus a three-person crew plus logistics)—tabulated in Supplementary Table S1. The conditional vendor-OR-gate-to-in-sample-candidate firing–rate ratio is invariant to this choice, because the per-CIP cash is common to numerator and denominator and cancels (Table S1); this is why only that invariant ratio and the conditional difference derived from it are carried in the main text, while the basis-sensitive replacement shares are confined to Table S1. We annualise each candidate firing rule over the any-train-running record (≈1.16 yr; the period during which at least one train is running, matching the deposited annualisation basis), value it at the per-CIP cash, and express the result as a share of one annualised full-array (72-element) membrane replacement (the dominant fouling cost [16]; annualised straight-line over an assumed array life, whose linear effect on the shares is stated in Table S1), computed on a consistent plant-total basis: both the CIP numerator (the plant-total firing rate, i.e., campaigns per year across all trains over the any-train-running record) and the replacement denominator (the full 72-element array) are plant-total, so shares are basis-consistent rather than mixing a per-train numerator with a plant-total denominator; the plant-total firing rate is the campaign count over that any-train-running record and is not further multiplied by the train count.

The per-CIP cash counts only chemicals and labour and is therefore uncertain in both directions: it omits the specific-energy and capacity value that timely cleaning restores (which understates cleaning’s value) and the per-CIP waste-neutralisation/COD, rinse-water and sampling overhead (which understates each cleaning’s true cost); because these omissions act oppositely, the net direction is undetermined and only an indexed comparison is reported. The vendor counterfactual is the full OR-gate (any of flow −10%, salt +10%, dP +15%) and its single legs, each evaluated on this site’s record. We report the observed direction (which rule produces more below-benchmark firings than the DP_norm operating point) as a retrospective descriptor and the magnitude as an indexed, basis-sensitive estimate; near-clean fractions are computed against the fixed commissioning baseline, whereas the vendor rules are natively per-CIP-reset, so a fully like-for-like re-evaluation of all three legs on per-CIP-reset baselines remains outside the present analysis and is treated as future work (Section 3.6).

Methods summary. Layer 1 compares recovery channels with five label-free retrospective checks. Layer 2 examines an endogenous in-sample candidate and reports below-benchmark firing separately. Layer 3 gates a Bayesian composite on identifiability, and the economic layer reports conditional indexed scenarios. Values regenerate from the deposited derived daily table and label-free CIP ledger.

## 3. Results

### 3.1. Analysis Units and Estimand Registry

Because the corpus is a single plant, every reported count is bound to an explicit unit of analysis, and the units are parallel views of one corpus rather than nested steps of a single funnel (Table 1). The 23 train-legs collapse to 18 within-train campaigns (≤7 d), of which 14 carry complete pre- and post-cleaning windows and ≈11 are effective-independent after an additional ≤3 d cross-train collapse; the label-free event-pair ledger (*n* = 17) and the 16-cell estimand grid (dataset × window × construction × placebo) are separate cross-sections of the same data, not subsets of the ≈11-event collapse. The cleaning ledger is label-free throughout: it contributes only date, train and chemical, never an operator effectiveness judgement.

Table 2 registers each target, outcome, window, comparison and analysis unit. Cleaning-recovery (E1) identifies DP_norm as the leading site-specific candidate; Bayesian identifiability (E2) leaves the joint four-weight vector unresolved while pinning only the salt passage marginal near zero. These are different targets evaluated on the same single-plant record, not independent replicates. Their numerical results remain site-specific.

### 3.2. A Label-Free Battery Identifies DP_norm as Leading Recovery Candidate

**Figure 2:**
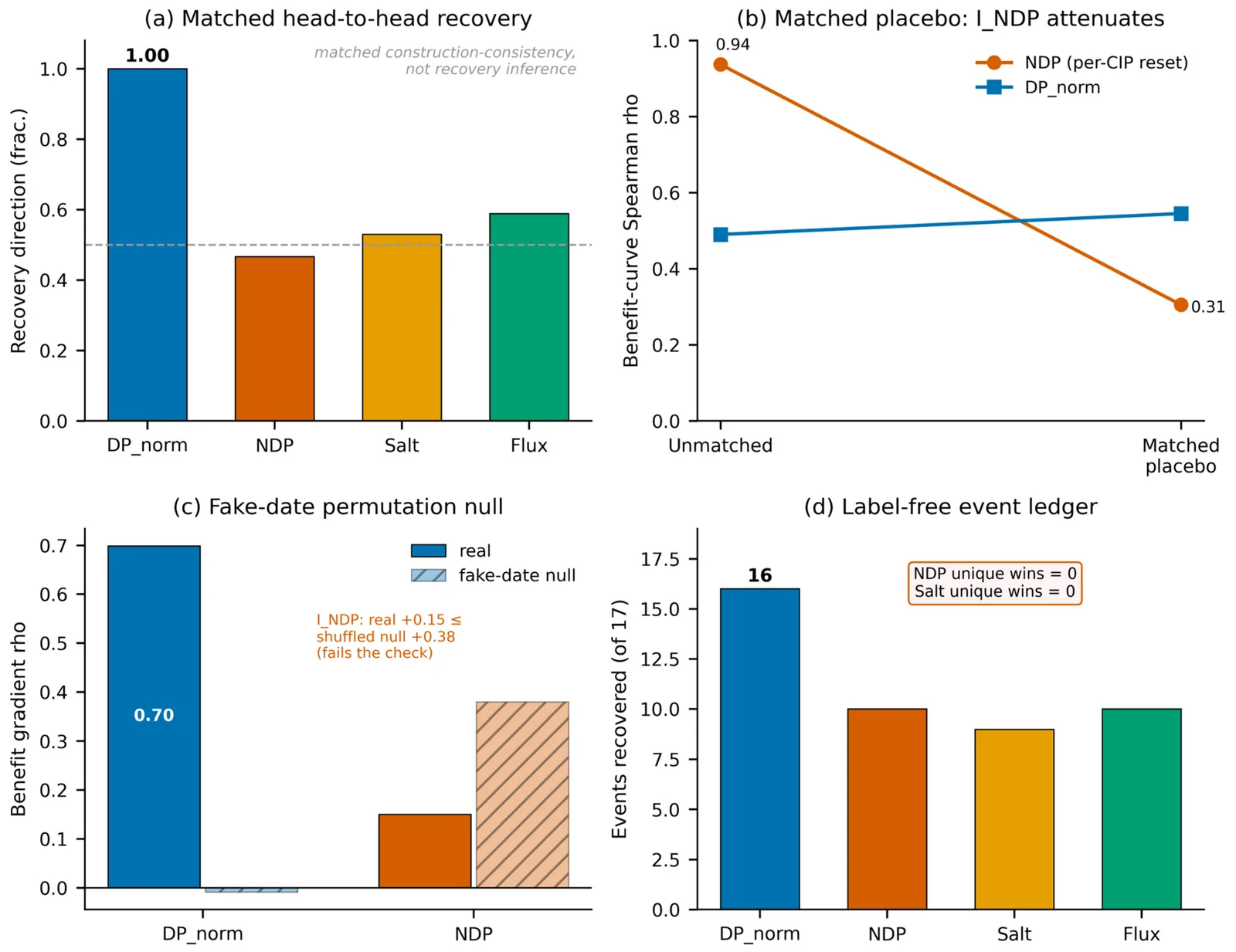
Retrospective validity battery comparing DP_norm and the per-CIP-reset NDP (I_NDP) candidate. (**a**) Across the 16-cell construction grid, DP_norm has recovery-direction score 1.00; train-leg and event-pair views are descriptive and carry no *p/q*-values. (**b**) Under the symmetric matched comparison, descriptive benefit-gradient correlations change from +0.46 to +0.55 for DP_norm and from +0.94 to +0.31 for I_NDP. (**c**) Under fake-date shuffling, DP_norm is +0.699 versus a shuffled median of ≈−0.009, while I_NDP is +0.150 versus +0.380; these are descriptive checks, not independent-unit inference. (**d**) DP_norm recovers in 16 of 17 event-pairs; the 16/17 and 21/23 counts are descriptive and pseudo-replicated. Recovery inference is confined to the nominal complete-campaign analysis (11/14; exact two-sided *p* ≈ 0.057; mid-*p* BH *q* ≈ 0.141), with ≈11 effective-independent events after collapse. I_NDP failed the matched and fake-date checks on this record; the pattern is consistent with reset/clip effects but does not identify a unique mechanism.

Layer 1 applies the same five retrospective checks to every channel (Figure 2; Table 3). DP_norm, computed against a fixed 2024 commissioning baseline, moved toward that baseline in 16 of 17 event-pairs and in all 16 construction-grid cells; these are descriptive consistency views rather than independent-unit inference. I_NDP showed recovery direction only in its native per-CIP-reset short-window cells, then attenuated under the symmetric matched comparison and was not distinguished from the fake-date null. It therefore failed the retrospective validity checks on this record; the pattern is consistent with reset/clip effects but does not identify a unique mechanism.

The construction-light cross-check showed the same direction at the raw-sensor level: un-normalised feed channel ΔP fell by a median of 0.020–0.033 bar, decreasing in 21 of 23 train-legs (91%) and in 16 of 17 event-pairs (94%). These train-leg and event-pair views are descriptive and pseudo-replicated; no *p*/*q*-value is assigned to them. Raw NDP, salt passage and permeability showed no repeatable post-cleaning movement. A decline led by feed channel pressure drop is consistent with the established mechanism that ΔP leads while salt passage and flux lag during RO fouling [2], reported at up to +218% per year on the same commercial seawater element class under heavy lead-element fouling [12].

The nominal complete-campaign analysis is the only recovery view carrying sign-test/FDR quantities. Among *n* = 14 complete-window campaigns, DP_norm recovered in 11 (79%; Wilson 95% CI [52%, 92%]), with exact two-sided sign-test *p* ≈ 0.057 and mid-*p* Benjamini–Hochberg *q* ≈ 0.141 (marginal and not FDR-significant); collapsing these campaigns leaves ≈11 effective-independent events. The same 11/14 count occurs at the ≤3/7/14 d collapse settings. DP_norm is therefore an operator-review candidate, not an autonomous or FDR-confirmed trigger. The 21/23 train-leg and 16/17 event-pair results remain descriptive and carry no *p*/*q*-values. Table 4 consolidates the per-channel results.

### 3.3. Two Falsification Tests Invalidate Per-CIP-Reset NDP Trigger

Two pre-specified retrospective checks assess sensitivity to regression-to-the-mean and chance date alignment (Figure 2b,c). Under the symmetric matched comparison, the DP_norm benefit–gradient correlation changed from ρ +0.46 to +0.55, whereas I_NDP changed from ρ +0.94 to +0.31; these matched-view correlations are descriptive rather than independent-unit inference. Under the fake-date check, the observed DP_norm gradient was +0.699 versus a shuffled-date median of ≈−0.009, while I_NDP was +0.150 versus +0.380. Thus I_NDP was not distinguished from its fake-date null. Across pooled longitudinal rows, I_NDP and fixed-baseline DP_norm had a descriptive Spearman ρ = −0.16; no row-level *p*-value is reported because serial dependence was not modelled. Together, these results show that the per-CIP-reset candidate failed the pre-specified validity checks on this record. The pattern is consistent with reset/clip effects, but the retrospective checks do not identify a unique mechanism or imply that NDP is globally uninformative. For this plant and this estimand, the per-CIP-reset NDP trigger is therefore invalidated as a clip-floor regression-to-the-mean artefact—a statement about what these data support here, not a claim that net driving pressure is globally uninformative, and not causal proof of a unique mechanism.

### 3.4. Exploratory, Endogenous, In-Sample Operator-Review Candidate

**Figure 3:**
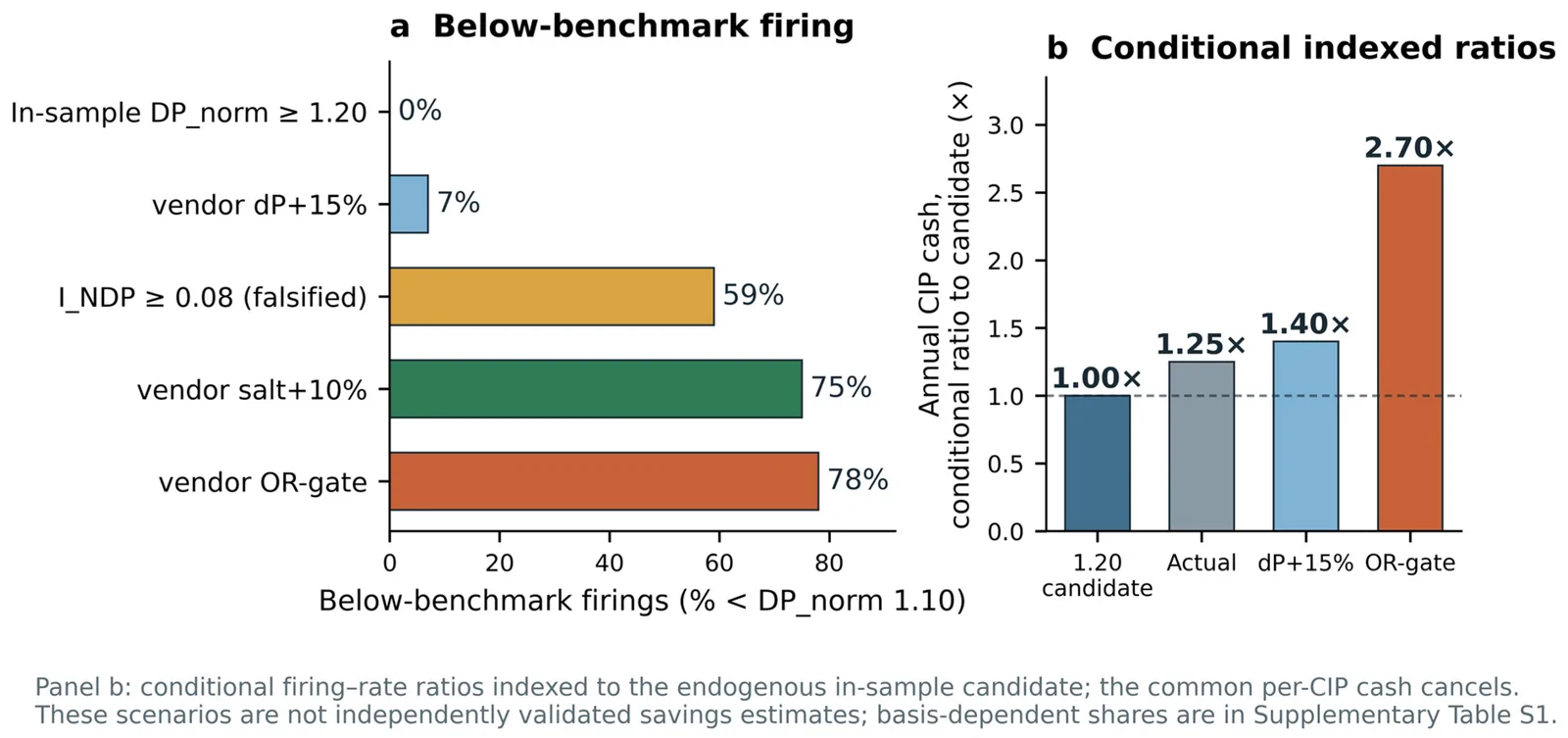
Retrospective Layer-2 descriptors and conditional indexed scenarios. (**a**) Below-benchmark firing (DP_norm < 1.10): in-sample DP_norm ≥ 1.20 candidate (≈0%, by construction), vendor dP + 15% leg (7%), per-CIP-reset I_NDP ≥ 0.08 candidate that failed validity checks (59%), vendor salt + 10% leg (75%) and full OR-gate (78%). (**b**) Conditional plant-total firing–rate ratios indexed to the endogenous in-sample candidate (1.00×, 1.25×, 1.40× and 2.70×). The common per-CIP cash cancels, while basis-dependent replacement shares remain in Supplementary Table S1. These ratios are exploratory scenarios, not independently validated savings estimates or evidence of equivalent hydraulic protection.

Layer 2 reports an exploratory, endogenous, in-sample DP_norm ≥ 1.20 candidate together with its retrospective below-benchmark firing descriptor (Figure 3; Table 5). On this site’s ≈1.16-yr any-train-running record, the vendor full OR-gate would fire ≈23.3 times yr^−1^ plant-total, with 78% of firings below DP_norm < 1.10; its salt-only and flow-only legs are below that benchmark on 75% and 74% of the occasions. The vendor dP + 15% leg fires ≈12.1 times yr^−1^, with 7% below benchmark. The in-sample 1.20 candidate has ≈0% below-benchmark firing by construction. These are retrospective targeting descriptors, not proof that a cleaning was unnecessary or that the candidate preserves equivalent protection.

The 1.20 candidate was chosen from the coarse τ-grid using duty and operator-log concordance: precision 0.70 (Wilson 95% CI [0.40, 0.89]), false-alarm rate 0.30, sensitivity 67%, ≈48 d median lead and 30% duty cycle. Lower candidates (1.10–1.15) are flagged 47–65% of the time, whereas 1.25–1.30 miss two-thirds of logged cleanings. Because operators already watch feed channel pressure, this in-sample selection and its reported concordance are endogenous and may overstate usefulness. The candidate is not a validated autonomous trigger.

Scope of the candidate review point. The campaign-collapse checks (≤3, ≤7 or ≤14 d) and baseline-free raw-sensor cross-check corroborate the recovery channel, not the numerical DP_norm ≥ 1.20 cut point. The τ-grid is coarse, and 1.20 was selected in-sample using duty and historical-log concordance; it is therefore exploratory and endogenous. Local sensitivity of the cut point and re-estimation under alternative fixed-baseline definitions or normalisation strategies were not evaluated. The point must be re-estimated prospectively and re-anchored whenever the baseline is re-established; TSB 106 absolute limits remain the overriding safety envelope [1].

### 3.5. Four-Weight Composite Is Non-Identifiable; Only Salt-Weight Marginal Contracts

**Figure 4:**
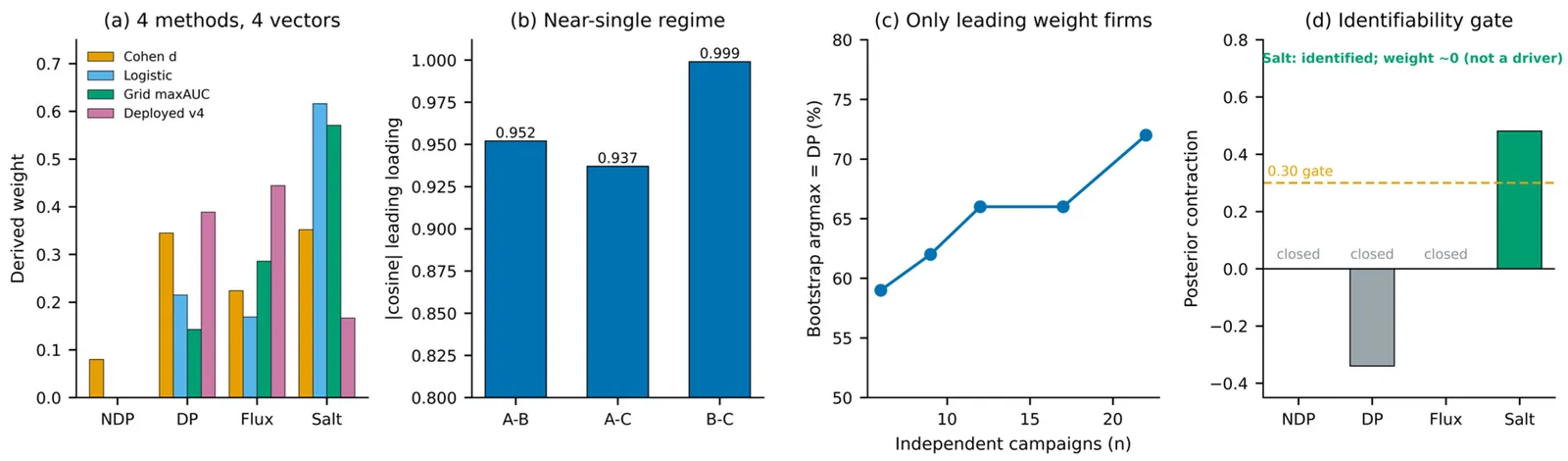
Layer 3, identifiability-gated Bayesian composite weighting on a single-regime plant. (**a**) Four different weighting methods (Cohen’s d, logistic, grid-search max-AUC, and the deployed v4 reference) return four different four-weight vectors, a direct symptom of non-identifiability. (**b**) Cross-train homogeneity: pairwise cosine similarity of the leading degeneration directions (A–B 0.952, A–C 0.937, B–C 0.999) with excitation rank ~ 1, indicating one fouling regime. (**c**) Leading-weight confidence: bootstrap argmax DP share rising from 59% (*n* = 6) to 72% (*n* = 22); only the leading weight firms with more campaigns. (**d**) Identifiability gate: posterior contraction per weight against the 0.30 contraction gate; only the salt passage weight clears it (contraction +0.481; posterior mean 0.043, 95% CI [0.001, 0.230]; identified as pinned near zero), while NDP, DP and flux fall below the gate and are not identified, so the mechanism reports a closed gate rather than imputing a fabricated vector. In (**d**), bar height is posterior contraction (identifiability), not channel importance: the salt passage weight is the sole identifiable weight and is pinned near zero (not a cleaning driver), and a negative value (DP) denotes a posterior wider than the prior (no information gained); the wide disagreement among the four methods in (**a**) is itself the non-identifiability. The joint four-weight vector is therefore non-identifiable; the daily severity ranking is nonetheless stable to the unresolved weights (rank Spearman 0.993; about 7% ambiguous days).

Layer 3 applies an identifiability-gated Bayesian power prior (Figure 4). The plant presents essentially one fouling regime: cross-train leading-direction cosines are 0.952, 0.937 and 0.999, and the stacked campaign design has excitation rank ≈1. The joint four-weight vector is therefore non-identifiable. Only the salt passage marginal clears the contraction gate, pinning its weight near zero (posterior mean 0.043, 95% CI [0.001, 0.230]); DP remains suggested, while NDP and flux retain the prior. Additional same-regime campaigns sharpen the leading coordinate but do not supply an orthogonal direction. The closed gates and unresolved coordinates are reported explicitly. The daily severity ranking remains stable to the unresolved weights (Spearman 0.993; ~7% ambiguous days), supporting a screening order with uncertainty but not an identified composite scale (Table 6).

### 3.6. Economic Layer: Observed Direction, Magnitude Basis-Sensitive

Table 5 gives a conditional scenario comparison against the vendor counterfactual. On a consistent plant-total basis, the OR-gate firing–rate ratio is ≈2.70× that of the endogenous, in-sample DP_norm ≥ 1.20 candidate; the difference is ≈1.7× the candidate’s annual CIP cash, or ≈63% of the OR-gate scenario. These ratios are invariant to the costing basis because common per-CIP cash cancels, but they inherit the candidate-selection endogeneity and are not validated savings. Relative to the historical operator log, the conditional difference is smaller. The OR-gate’s below-benchmark firing is driven by its salt/flow legs rather than its dP + 15% leg. Because the benchmark is fixed-baseline whereas vendor rules are natively per-CIP-reset, a like-for-like re-evaluation is required before interpreting the magnitude as vendor over-firing.

In plain operational terms, this conditional retrospective scenario compares ≈23.3 OR-gate firings yr^−1^ with ≈8.6 firings yr^−1^ for the in-sample candidate, a ≈63% difference. All candidate firings occurred above the retrospective DP_norm benchmark; this does not establish equivalent hydraulic protection, safety or avoided damage. Current practice is closer to the candidate (ratio ≈ 1.25×), but neither comparison is a validated saving.

Results summary. DP_norm is the leading recovery candidate, but nominal complete-campaign recovery is marginal and not FDR-significant (11/14; *q* ≈ 0.141). I_NDP fails the retrospective validity checks, with a pattern consistent with reset/clip effects. The 1.20 point and ratios indexed to it are exploratory, endogenous and site-specific. The four-weight composite is non-identifiable except for the near-zero salt marginal.

## 4. Discussion

### 4.1. Principal Contribution: Methodological and Cautionary Result

The contribution is methodological and cautionary. First, the estimand registry defines the target, while registered campaigns/events/days/trains remain the observational units and effective-independent campaigns/events carry recovery inference. Second, the retrospective battery compares candidate recovery channels without operator-effectiveness labels. Third, identifiability gating reports when composite weights remain unresolved. Fourth, the falsification of the per-CIP-reset I_NDP trigger: it collapses under the pre-specified matched placebo and is reproduced by the fake-date null on this record—the pattern a clip-floor regression-to-the-mean artefact predicts—a transferable warning that per-cleaning-reset, clipped indicators can manufacture apparent recovery by construction, while not identifying a unique mechanism. DP_norm remains an operator-review candidate rather than a confirmed trigger (11/14; exact two-sided *p* ≈ 0.057; mid-*p* BH q ≈ 0.141). The numerical 1.20 point is an exploratory, endogenous, in-sample heuristic.

Evidential status of each claim. The I_NDP results are descriptive retrospective patterns: attenuation after matching, failure to exceed the fake-date summary and a pooled descriptive association of ρ = −0.16 with fixed-baseline DP_norm; no pooled-row *p*-value is used. DP_norm recovery is directionally consistent but not FDR-confirmed (11/14; exact two-sided *p* ≈ 0.057; mid-*p* BH *q* ≈ 0.141) and is presented only for operator-review. The DP_norm ≥ 1.20 point, its log-concordance and lead-time measures, and ratios indexed to it are exploratory, endogenous and site-specific.

An estimand specifies the target; it is not an observational or inferential unit. Campaigns and events remain the analysis units, and the single plant defines the scope of inference. Different operational questions can yield different channel summaries from the same archive, but those estimands are not independent plant replicates. Repeated within-plant observations support site-specific estimation and do not relax the need for cross-plant validation.

### 4.2. DP_Norm Is Decision Support, Not an Autonomous Trigger

DP_norm was the leading recovery candidate on the retrospective construction grid, but the independent-unit evidence remains limited. The 16/17 event-pair and 21/23 train-leg views are descriptive and carry no *p*/*q*-values. On the nominal complete-campaign analysis, recovery was 11/14 with exact two-sided *p* ≈ 0.057 and mid-*p* BH *q* ≈ 0.141; collapsing yields ≈11 effective-independent events. DP_norm is therefore an operator-review candidate, not an autonomous or FDR-confirmed trigger. The Layer-2 DP_norm ≥ 1.20 point is an exploratory, endogenous, in-sample heuristic that requires prospective re-estimation and validation.

### 4.3. Why Per-CIP-Reset NDP Trigger Fails

The per-CIP-reset I_NDP candidate attenuated after the symmetric matched comparison (ρ +0.94 → +0.31) was not distinguished from the fake-date summary (+0.150 versus +0.380), and had a descriptive pooled association of ρ = −0.16 with fixed-baseline DP_norm; no pooled-row *p*-value is reported. It therefore failed the retrospective validity checks on this record and is invalidated, for this plant and estimand, as a clip-floor regression-to-the-mean artefact. The pattern is consistent with reset/clip effects: resetting the post-cleaning baseline mechanically forces a clipped loss index toward zero and can create apparent pre-to-post improvement. These checks do not identify a unique mechanism or globally refute NDP. Per-cleaning-reset clipped indices should remain unvalidated as standalone CIP timers until tested against raw-sensor, matched placebo and fake-date checks on independent data.

### 4.4. Identifiability: Reporting Closed Composite Gate

With one fouling regime, the joint composite is non-identifiable and only the salt passage marginal clears the gates (Section 3.5). The gated power prior reports the remaining gates as closed and retains the prior until multi-plant or multi-regime data provide additional excitation. The daily severity ranking remains stable, so this layer supports a screening order with an uncertainty envelope but not an identified composite scale or evidence of outperformance over the vendor rule.

### 4.5. Economics: Observed Direction, Magnitude Basis-Sensitive, Cash Not Ageing

Fouling cost in full-scale RO includes early membrane replacement and cleaning [16,17]. On this record, salt-only and flow-only OR-gate firings were below the fixed-baseline hydraulic benchmark on 75% and 74% of the occasions, versus 7% for the vendor dP + 15% leg; this comparison remains pending for a per-CIP-reset like-for-like re-evaluation. The directly measured cost category is CIP cash (chemicals and labour), not membrane ageing. Non-oxidative cleaning has been reported not to degrade virgin polyamide under the tested coupon conditions [18], but this record contains no replacement event or autopsy, so no life-extension benefit is claimed. Conditional annual cash for another site can be written as (R_rule − R_candidate) × C_CIP. Because R_candidate here was selected endogenously in-sample, the resulting ratios are exploratory scenarios rather than validated savings.

### 4.6. Comparison with Prior Work and Within-Plant Data-Window Flip

Data-driven CIP optimisation has been demonstrated on full-scale RO [5], including the authors’ related studies of cleaning protocols [19] and energy and pretreatment [20]. Broader work includes an AI fouling-prediction review [7], digital-twin restoration planning [8] and supervised seawater-RO fouling prediction [9]. PCA-family RO monitoring has also been used without operator cleaning effectiveness labels: UPCA accommodates operating-mode and cleaning-cycle changes in RO desalination [21], and KPCA detects process deviations in pharmaceutical RO [22]. These studies address prediction, fault detection or anomaly monitoring. The present analysis instead pre-specifies a post-CIP recovery target and checks whether candidate signal construction affects apparent recovery. This comparison is limited to the cited set and is not a systematic review of label-free or unsupervised SCADA methods; no priority claim over such methods is made.

An earlier, shorter-window analysis of this plant favoured NDP, whereas the present longer window identifies DP_norm as the leading candidate and finds that per-CIP-reset I_NDP fails the retrospective validity checks. This within-plant change is evidence that channel selection depends on the analysis window and construction.

### 4.7. Practical Implications for Operators

For a field operator, the workflow compares three generic single-indicator thresholds with a plant-calibrated review aid (Box 1). The vendor dP + 15% leg is close to the retrospective DP_norm benchmark. Salt/flow legs remain unvalidated as standalone timers at this site, and the manufacturer safety envelope remains unchanged.

Box 1 **Operator-review decision aid. Every step is a human review or logging action, not automated control.**

1. Monitor DP_norm on a fixed commissioning baseline, with raw feed channel dP tracked in parallel.

2. Review flag: raise a review (not a clean/no-clean) flag when DP_norm ≥ 1.20 (≈48 d median lead; ≈0% below the hydraulic benchmark DP_norm < 1.10 on this record).

3. Operator confirmation: before any action, confirm the crossing against trend, operating mode, and permeate (product–water) quality.

4. Hard override: the absolute differential pressure safety limits (TSB 106: 1 bar per element, 3.8 bar per housing) supersede any ranking-based decision support [1].

5. Log each crossing prospectively, together with the operator action taken and the observed recovery.

6. Re-estimate the gates and operating point after each effective-independent campaign.

The workflow is computable in real time—every quantity is causal (trailing medians, a fixed baseline, no look-ahead) and derives from standard historian tags—but it has been validated here only retrospectively. Deployment is therefore specified as a review flag inside the existing SCADA/HMI alarm layer with mandatory operator confirmation (Box 1), and the prospective crossing log (Box 1, steps 5–6) is the mechanism by which real-time use accrues the prospective evidence a deployable trigger would require. In the same integration, the workflow serves as a signal-selection and verification layer beneath model-based tools such as digital twins and machine-learning predictors [7,8]: it provides a retrospectively screened candidate channel for prospective validation before such tools are trained on it.

### 4.8. Limitations, Threats to Validity, and Future Work

The evidence is bounded. All analyses are retrospective and in-sample, with no randomised control, prospective hold-out or independent-plant validation; recovery remains under-powered (*q* = 0.141 at ≈11 effective-independent events after collapse). The endpoint is hydraulic only. The near-single regime limits both composite identification and external validity. No independent pressure-sensor drift audit based on zero/span records, redundant sensors or a pre-specified drift threshold was available. The raw-sensor check is baseline-free but sensor-dependent, and fake dates address alignment rather than drift. The 1.20 candidate was selected partly against the endogenous operator log; threshold sensitivity under alternative fixed baselines or normalisation was not evaluated, and all indexed economics inherit that endogeneity. Future work requires pre-registered prospective campaign-level validation, alternative-baseline threshold re-estimation, pressure-calibration auditing, and multi-plant/multi-regime data. Future studies should also collect per-CIP foulant-chemistry or autopsy data and evaluate the workflow across membrane types, feed-water regimes, operating conditions and plant scales.

## 5. Conclusions

For operators, DP_norm ≥ 1.20 is only an exploratory, in-sample review flag; its ≈48 d median lead, ≈0% below-benchmark fraction and historical-log concordance are retrospective and endogenous. Nominal complete-campaign recovery remains marginal and not FDR-significant (11/14; exact two-sided *p* ≈ 0.057; mid-*p* BH q ≈ 0.141), so DP_norm is an operator-review candidate, not an autonomous or FDR-confirmed trigger. The 16/17 event-pair and 21/23 train-leg results are descriptive pseudo-replicated counts and carry no *p/q*-values. I_NDP failed the matched and fake-date validity checks and is invalidated on this record as a clip-floor regression-to-the-mean artefact—bounded to this plant and estimand, not a global refutation of NDP. The estimand defines the target, campaigns/events remain the analysis units, and the single plant remains the scope of inference. The joint composite is non-identifiable except for the near-zero salt marginal. Ratios indexed to the in-sample candidate are conditional scenarios, not validated savings or evidence of equivalent protection. TSB 106 limits remain hard overrides.

## Figures and Tables

**Table 1:** Analysis-unit crosswalk (branched, not a funnel): each analysis, its unit of analysis, and its count.

Analysis/Object	Unit of Analysis	Count	Role
CIP ledger legs	train-leg (acid/alkaline stage)	23	descriptive only; pseudo-replicated (one physical cleaning contributes several overlapping legs, so legs are not independent samples)—never carries power/significance
Within-train campaigns	campaign (same-train-legs ≤ 7 d collapsed)	18 (A: 3, B: 8, C: 7)	campaign definition
Complete-window campaigns	campaign with full pre + post windows	14	matched event-study recovery (primary inferential unit)
Effective-independent events	plant-wide event (+≤3 d cross-train collapse)	≈11	conservative effective n for power/FDR
Label-free event-pair ledger	event-pair	17	head-to-head construction grid; raw-sensor descriptive count
Estimand grid	cell (dataset × window × construction × placebo)	16	non-circularity/construction-robustness of selection
FDR-effective n	—	≈11–14	all multiplicity, power, and FDR statements

Note: views are not nested; e.g., the *n* = 17 event-pair ledger is not a subset step of the ≈11 effective-independent collapse.

**Table 2:** Estimand registry: outcome, window, comparison, unit, selected/identifiable channel, and status in this work.

Estimand	Operational Question	Outcome/Window/Comparison/Unit	Selected or Identifiable Channel	Status in This Work
E1	CIP cleaning-recovery/operator decision support (does a cleaning recover the channel; when to review)	Post-CIP recovery vs. matched placebo; ±14 d; effective-independent campaign	DP_norm (feed channel hydraulic)	This work: leading operator-review candidate; not autonomous, not FDR-confirmed (11/14 = 79%, exact two-sided sign-test *p* ≈ 0.057, mid-*p* BH *q* ≈ 0.141; ≈11 effective-independent events). Per-CIP-reset NDP (I_NDP) failed the retrospective validity checks; its pattern is consistent with reset/clip effects
E2	Bayesian identifiability of a composite weight (which weight can the data learn)	Posterior contraction (>0.30 gate) under DP-dominant power prior; per-CIP baseline; day-rows	Salt passage weight is the only marginal that contracts, pinned ≈0 (posterior mean 0.043 [0.001, 0.230]; contraction +0.481). Joint four-weight vector non-identifiable	This work: native identifiability-gating result; “identified” = pinned near-zero, not a cleaning driver

**Table 3:** Retrospective validity battery: five label-free checks selecting DP_norm and invalidating the per-CIP-reset NDP (I_NDP) trigger.

Retrospective Check (Unit)	DP_norm (Leading Candidate)	I_NDP (Per-CIP-Reset NDP Loss)	Salt Passage/Specific Flux
1. Matched head-to-head across 16-cell construction grid (recovery direction)	moves toward baseline in 16/16 cells (recover_dir = 1.00; descriptive construction consistency) †	recovery direction only in native per-CIP-reset short-window cells; fails the retrospective validity checks (Tests 2–3)	inconsistent across cells
2. Symmetric matched (level + slope) comparison—benefit-gradient ρ (pre → post matching)	+0.46 → +0.55 (descriptive matched-view correlation)	+0.94 → +0.31 (descriptive; attenuated after matching)	not selected
3. Fake-date comparison—observed gradient vs. shuffled summary	observed +0.699 versus shuffled median ≈ −0.009 (descriptive)	+0.150 versus +0.380 (not distinguished from the fake-date null; descriptive)	not selected
4. Raw-sensor physical cross-check	raw feed ΔP falls, median −0.020 to −0.033 bar; 21/23 legs (91%), 16/17 pairs (94%); descriptive pseudo-replicated counts, no *p*/*q*-value	no repeatable movement	no repeatable movement
5. Retrospective below-benchmark firing (DP_norm < 1.10)	in-sample DP_norm ≥ 1.20 candidate: ≈0% (by construction)	58.6% (48–55% even floor-free)	salt-only 75%; flow-only 74%
Nominal complete-campaign recovery (*n* = 14; ≈11 effective-independent)	11/14 (79%); exact two-sided sign-test *p* ≈ 0.057; mid-*p* BH *q* ≈ 0.141 (marginal, not FDR-significant)	50%	flux 50%; salt 57%

† The recover_dir = 1.00 result is a descriptive construction-consistency count; no *p*/q-value is assigned to the 17-pair or matched-view analyses. Recovery inference is anchored to the nominal complete-campaign row, with ≈11 effective-independent events after collapse. The per-CIP clip-based benefit curve is excluded as a selection test because a zero-recovery floor-clip null produces ρ ≈ +0.66 [0.43, 0.85]; the observed I_NDP value (+0.951) is not by itself recovery evidence.

**Table 4:** Performance summary of the four normalised membrane channels across the three layers (quantities defined in Section 2; recovery inference is confined to the nominal complete-campaign analysis, with ≈11 effective-independent events after collapse).

Channel	Layer 1: Recovery Direction (16-Cell Grid)	Campaign-Level Recovery (*n* = 14)	mid-*p* BH q	Retrospective Validity-Check Result	Layer 2: Below-Benchmark Firing (DP_norm < 1.10)	Layer 3: Identifiability
DP_norm (fixed baseline)	moves toward baseline in 16/16 descriptive construction-grid cells	11/14 (79%; Wilson [52%, 92%])	0.141 (marginal, not FDR-significant)	matching correlation remained similar; observed fake-date gradient exceeded shuffled median (descriptive)	≈0% at the in-sample DP_norm ≥ 1.20 candidate (by construction)	joint weight not identified (“suggested”; gate closed)
NDP (per-CIP-reset I_NDP)	recovery direction only in native per-CIP-reset cells	50%	≈0.95	failed on this record—attenuated after matching; not distinguished from fake-date null	58.6% (48–55% even floor-free)	rides the prior (gate closed)
Specific flux (25 °C)	inconsistent across cells	50%	≈0.95	not selected	vendor flow-only leg: 74%	rides the prior (gate closed)
Salt passage (25 °C)	inconsistent across cells	57%	≈0.95	not selected	vendor salt-only leg: 75%	only contracting marginal, pinned ≈0 (0.043 [0.001, 0.230])

**Table 5:** Retrospective below-benchmark firing and conditional indexed cost ratio by firing rule (evaluated on this site’s ≈1.16-yr any-train-running record). Basis-dependent replacement shares are given in Supplementary Table S1.

Firing Rule	Annualised CIP Rate, Plant-Total (yr^−1^)	Firings Below Hydraulic Benchmark (DP_norm < 1.10)	Median DP_norm at Firing	Conditional CIP Cash Ratio to In-Sample Candidate (×)
In-sample DP_norm ≥ 1.20 candidate (operator-review)	8.6	≈0% (by construction)	≥1.20	1.00×
Operator actual (historical log)	10.8	≈8%	—	1.25×
Vendor dP +15% leg (hydraulic)	12.1	7%	1.156	1.40×
Vendor full OR-gate (flow −10% OR salt +10% OR dP +15%)	23.3	78%	0.958	2.70×

Notes: All economic quantities are indexed, never currency. The final column is a conditional plant-total firing–rate ratio to the in-sample candidate; the common per-CIP cash cancels, so the ratio is invariant to the costing basis. Replacement shares remain basis- and assumed-life-sensitive and are confined to Supplementary Table S1. Ratios use unrounded annualised cash. Near-clean fractions use the fixed commissioning baseline, whereas the manufacturer rules are natively per-CIP-reset; a like-for-like re-evaluation remains required. Because the reference candidate was selected endogenously in-sample, these ratios are conditional scenarios, not independently validated savings estimates.

**Table 6:** What single-plant N resolves, and what it does not.

Method Element	Layer	Sharpens with Single-Plant N?	Evidence/Bottleneck
Indicator screening (which channel is the leading recovery candidate)	1	Directionally consistent on this record; confirmation remains limited	DP_norm moves toward baseline in 16/16 descriptive construction-grid cells; raw feed ΔP falls in 21/23 descriptive legs
Candidate threshold + retrospective descriptors	2	Yes—per campaign	endogenous in-sample DP_norm ≥ 1.20 candidate; ≈0% below benchmark; ≈48 d median lead; 30% duty
Leading-weight confidence (DP share)	3	Yes—leading coordinate only	bootstrap argmax DP rises 59% (*n* = 6) → 72% (*n* = 22); leave-one-train-out still flips NDP↔DP
FDR confirmation of recovery	1→2	Yes—directional now, confirmation later	mid-*p* BH *q* = 0.141 (*n* ≈ 11–14); ≈25+ effective-independent campaigns projected for FDR < 0.05
Full four-weight composite vector	3	No—needs multi-plant/multi-regime	cross-train cosine 0.952/0.937/0.999; excitation rank ≈ 1

## Data Availability

The derived data and analysis code required to reproduce the principal tables and figures in this article are openly available in the Zenodo repository at https://doi.org/10.5281/zenodo.21003996, version-tagged to processing pipeline “009”. The deposit comprises: a de-identified derived daily train-level indicator table (fixed-baseline, temperature-corrected DP_norm, net driving pressure, 25 °C specific flux and 25 °C salt passage, together with the un-normalised feed channel differential pressure and the per-CIP-reset constructions); an anonymised label-free clean-in-place (CIP) campaign table (relative date, train, and acid/alkaline stage only); the pre/post and placebo window definitions with fixed random seeds; the fake-date permutation null outputs; Table 3 and Table 5 regeneration scripts (and the scripts for all remaining tables and figures); a SHA-256 raw data hash manifest; a column dictionary; and the pinned software environment. All economic quantities are deposited only as dimensionless ratios, multiples and shares; no procurement prices, no currency values, no membrane element model or membrane area, and no information identifying the plant owner are included. The raw 10 min supervisory control and data acquisition (SCADA) series underlying the derived table are not publicly released, for reasons of operational confidentiality and ongoing legal proceedings, and because the plant owner (a public water utility) is not named in this work. The derived daily table is an irreversible aggregation of the raw series; together with the deposited analysis outputs and the figure- and table-regeneration code it is sufficient to regenerate the reported table and figure values from the deposited derived outputs. The upstream scripts that create those derived outputs additionally read the withheld raw 10 min series (hashes provided), so the raw series are not required to re-render the submitted tables and figures, but are required to audit the upstream derivation from the raw data. The raw series are available to the editor and peer reviewers, under a confidential data-use agreement, on request. Every headline value traces to a re-runnable computation on the full archived dataset rather than an inherited or hard-coded constant.

## Supplementary Materials

The following supporting information can be downloaded at https://www.mdpi.com/article/10.3390/membranes16080257/s1, Table S1: annualised CIP cash expressed as a share of annualised full-array (72-element) membrane replacement, tabulated under two explicitly labelled cost bases (an “accounting basis” and a “full-operation basis”), together with the one-CIP-versus-element cost ratios; the table also records that the conditional OR-gate-to-in-sample-candidate 2.70× firing–rate ratio is invariant to the chosen basis (the per-CIP cash cancels). All supplementary quantities are indexed (multiples and shares); no currency values are given. The de-identified derived daily indicator table, the label-free CIP ledger, and the table- and figure-regeneration codes are openly archived at Zenodo (concept DOI: https://doi.org/10.5281/zenodo.21003996).
