# Supplementary material for: Auditable Clean-in-Place Decision Support from Routine SWRO SCADA: Selecting Differential-Pressure Recovery and Falsifying a Per-CIP-Reset Trigger
Source: Membranes (Basel). 2026 Jul 28;16(8):257. doi: 10.3390/membranes16080257 (PMC13515404; doi:10.3390/membranes16080257)
Supplement: Supplementary file 1 [file membranes-16-00257-s001.zip › membranes-4448411-supplementary.pdf]

Supplementary Materials

Auditable Clean-in-Place Decision Support from Routine SWRO SCADA: Selecting Differential-Pressure Recovery and Falsifying a Per-CIP-Reset Trigger

Yi Hsiang Su, Fan Cheng Meng and Pieh Yu Chang

This file contains Supplementary Table S1 (two-basis sensitivity of the indexed cleaning-cost comparison). All quantities are dimensionless (ratios, multiples, and shares); no procurement prices, per-m^3 figures, or currency values are disclosed. The derived data and code that regenerate the firing rates underlying this table are in the Zenodo deposit (concept DOI 10.5281/zenodo.21003996), version-tagged to processing pipeline "009".

Table S1. Two-basis sensitivity of the indexed cleaning-cost comparison

The per-CIP cash at this plant is dominated by chemicals (EDTA-dominant) and labour, with net production loss ≈0 because two of the three trains meet routine demand (main text §2.1). Because the labour and logistics component can be scoped in more than one defensible way, all absolute-magnitude quantities are reported under two labelled costing bases:

- **Accounting basis** — chemicals (EDTA-dominant) plus single-operator attended labour (the CIP is absorbed within the running shift; net production loss ≈0 owing to train redundancy). These values reproduce the accounting-basis shares in Part A; the main-text Table 5 conditional ratio remains basis-invariant because the common per-CIP cash cancels.

- **Full-operation basis** — chemicals plus a three-person crew plus logistics; a fuller, deliberately conservative scoping of the same CIP. On this basis the per-CIP cash is ≈2.18× the accounting per-CIP cash.

Neither basis is offered as *the* cost; they bound the labour/logistics scope. Every quantity below is indexed and carries no currency.

Part A — Annualised CIP cash as a share (%) of one annualised full-array (72-element) membrane replacement, by firing rule and costing basis.

Firing rule	Plant-total CIP rate (yr^{-1})	Conditional ratio to in-sample candidate (×) [basis-invariant]	Share — accounting basis (%)	Share — full-operation basis (%)
In-sample DP_norm ≥ 1.20 candidate (operator review)	8.6	1.00	18.3	39.9

Operator actual (historical log)	10.8	1.25	23.0	50.1
Vendor dP +15% leg (hydraulic)	12.1	1.40	25.7	56.2
Vendor full OR-gate (flow −10% OR salt +10% OR dP +15%)	23.3	2.70	49.7	108.1

Part B — Per-element indexed cost multiples, by costing basis. One train-CIP cleans the ≈24 elements of that train (three pressure vessels × eight elements). The one-CIP multiple is displayed to two decimals; the per-element-cleaned multiple is 24 ÷ the *unrounded* one-CIP value, so the accounting basis reconciles to 78× (not 24/0.31 = 77) and the full-operation basis to 36×.

Quantity	Accounting basis	Full-operation basis
One train-CIP cash ÷ price of one replacement element	0.31×	0.67×
Price of one replacement element ÷ per-element cleaning share (≈24 ÷ the one-CIP multiple above)	78×	36×

Notes.

1. **Basis-invariance and scope of the ratios.** Any firing-rule ratio is invariant to the costing basis because the common per-CIP cash cancels. Ratios use unrounded plant-total rates, so displayed one-decimal values reproduce them only to rounding. The OR-gate/in-sample-candidate ratio is **2.70×** on both bases; operator/candidate and vendor-dP/candidate ratios are **1.25×** and **1.40×**. The conditional OR-gate-to-candidate difference is **≈1.7×** the candidate's cash or **≈63%** of the OR-gate scenario. The DP_norm ≥ 1.20 reference candidate was selected endogenously in-sample; these are conditional scenario ratios, not independently validated savings estimates.

2. **Basis scaling.** The full-operation per-CIP cash is ≈2.18× the accounting per-CIP cash (from unrounded values; the one-decimal displayed shares and multiples reproduce this to rounding, e.g. 39.9/18.3 = 2.18, 108.1/49.7 = 2.18, 0.67/0.31 = 2.16), so every Part-A share and the one-CIP-vs-element multiple in Part B scale up by ≈2.18× between bases, while the per-element-cleaned multiple scales down correspondingly (78× → 36×). Under the tabulated full-operation assumptions, the OR-gate's annual cleaning cash slightly exceeds one full annualised array replacement (108.1%); this is the same conditional scenario comparison summarised by the invariant 2.70× firing-rate ratio.

3. **Array-life assumption.** Part-A shares annualise the 72-element replacement straight-line over an assumed array life (5 yr in the tabulated values) and scale linearly with that assumption: a longer assumed life lowers the annualised replacement denominator and raises every share proportionally, on both bases. The ratios in note 1 are unaffected by the array-life assumption (it cancels).

4. **Plant-total consistency.** Shares use a consistent plant-total basis: the CIP numerator is the plant-total firing rate (campaigns per year across all trains over the ≈1.16-yr any-train-running record) and the replacement denominator is the full 72-element array; the numerator is not further multiplied by the train count.

5. **Provenance and directionality.** The per-CIP cash is documented internally from the plant CIP recipe and local rates; it is **not** derived from any external per-m^3 chemical figure. It counts only chemicals and labour and is uncertain in both directions (it omits the specific-energy/capacity value that timely cleaning restores and the per-CIP waste-neutralisation/COD, rinse-water and sampling overhead), so only indexed, basis-sensitive comparisons are reported. Near-clean fractions in main-text Table 5 are computed against the fixed 2024 commissioning baseline, whereas the vendor rules are natively per-CIP-reset; a like-for-like re-evaluation on per-CIP-reset baselines is required before the vendor rule is read as over-firing (main text §3.6, §4.5).
